# Vincamine exerts hepato-protective activity during colon ligation puncture-induced sepsis by modulating oxidative stress, apoptosis, and TNFα/Nrf-2/Keap-1 signaling pathways

**DOI:** 10.1038/s41598-024-69729-1

**Published:** 2024-08-23

**Authors:** Rania Alaaeldin, Reham H. Mohyeldin, Ehab E. Sharata, Mina Ezzat Attya, Moustafa Fathy

**Affiliations:** 1 Department of Biochemistry, Faculty of Pharmacy, Deraya University, Minia, 61111 Egypt; 2 Department of Pharmacology and Toxicology, Faculty of Pharmacy, Deraya University, Minia, 61111 Egypt; 3 Department of Pathology, Faculty of Medicine, Minia University, Minia, 61519 Egypt; 4 Department of Biochemistry, Faculty of Pharmacy, Minia University, Minia, 61519 Egypt

**Keywords:** Vincamine, CLP, Hepatic injury, TNFα, Cleaved caspase 3, Nrf-2, Keap-1, Diseases, Gastroenterology, Molecular medicine

## Abstract

Sepsis is a pathological and biochemical disorder induced by numerous infections, leading to critical illness and a high mortality rate worldwide. Vincamine is an indole alkaloid compound obtained from the leaves of Vinca minor. The present study aims to investigate the hepato-protective activity of vincamine during colon ligation puncture (CLP)-induced sepsis at the molecular level. Sepsis was induced using the CLP model. Liver function enzymes such as ALT and AST were analyzed. The hepatic antioxidant status (SOD and GSH), lipid peroxidation (MDA), the pro-inflammatory cytokines (TNFα, IL-6, and IL-1β), bax, bcl2, and cleaved caspase 3 proteins were estimated. Nrf-2 and Keap-1 protein expression was evaluated using western blotting. Histopathological investigation of liver tissues was also performed. CLP-induced sepsis led to liver injury through the elevation of ALT and AST liver enzymes. Oxidative stress was initiated during CLP via the suppression of hepatic GSH content and SOD activity and the elevation of MDA. The inflammatory condition was activated by the upregulation of TNFα, IL-6, IL-1β, and Keap-1 and the downregulation of Nrf-2 proteins. The apoptosis was initiated through the activation of bax and cleaved caspase 3 protein expression and inhibition of bcl2 protein expression. However, vincamine significantly improved the hepatic histological abnormalities and decreased liver enzymes (ALT and AST). It ameliorated oxidative stress, as evidenced by reducing the hepatic MDA content and increasing the SOD activity and GSH content. Moreover, vincamine reduced the hepatic content of TNFα, IL-6, IL-1β, and Keap-1 and increased Nrf-2 protein expression. Additionally, it upregulated bcl2 protein expression and downregulated bax and cleaved caspase 3 protein expression. Vincamine exhibited hepato-protective potential during CLP-induced sepsis via the cross-connection of antioxidant, anti-inflammatory, and anti-apoptotic activities by modulating TNFα/IL-6/IL-1β/Nrf-2/Keap-1 and regulating bax/bcl2/cleaved caspase 3 signaling pathways.

## Introduction

Sepsis is a dysregulated systemic inflammatory reaction to infection and is considered a primary cause of death among hospitalized patients in intensive care units (ICUs)^1^. Septic cases have a high mortality rate of 28%-48% and a global incidence of nearly 18 million cases yearly, usually caused by a deficient human response to infection^2^. The progression of the disease starts with inflammation, then sepsis and septic shock, followed by numerous organ failures^3^. Infants, elderly, cancerous, and immune-weekend patients are the most prone to sepsis and septic-related death^4^. The occurrence of sepsis is predicted to increase by 1.5% annually due to the increasing number of elderly patients^5^.

The cecal-ligation puncture (CLP) method is frequently used to induce sepsis in animals. Puncturing the cecum, which is filled with bacteria, would release bacteria into the systemic circulation and cause multiple-organ dysfunction, septic shock, and eventually death^6^. The CLP model is considered an acceptable procedure to induce sepsis more accurately than other techniques such as injecting purified bacteria or endotoxins^7^.

The pathological inflammatory condition of sepsis leading to death is not fully understood, especially for crucial organs such as the liver. Liver injury frequently arises in the early stages of sepsis, and it is a key promoter for multiple organ damage with subsequent sepsis-induced death^3^. The frequency of liver dysfunction and liver failure during sepsis is 34–46% and 1.3–22%, respectively^8^.

The management of sepsis usually includes the administration of antibiotics, fluid support, and the removal of infectious agents. Although these interventions have shown fair outcomes in lowering mortality rates, managing the triggered, uncontrolled inflammatory response is still urgently needed^9^.

Natural products containing indole-alkaloids have shown several pharmacological activities, such as antioxidant, anti-inflammatory, antimicrobial, and anti-tumor activity. Vinca minor’s leaves have shown profound antioxidant and antihyperlipidemic activities^10,11^. Specifically, vincamine, which is a monoterpenoid indole alkaloid compound extracted initially from Vinca minor’s leaves, is an important medicinal plant that exhibits multiple bioactivities, including antidiabetic, antihypertensive, antioxidant, antibacterial, and anti-tumor activities^12^. Moreover, the potential antioxidant activity of vincamine has been studied. Vincamine ameliorated inflammation activated by lipopolysaccharide in human corneal epithelial cells^13^. Also, it, in addition to zafirlukast, protected the liver via relieving tamoxifen-induced oxidative stress and liver injury^10^. Additionally, it has shown anti-inflammatory and anti-oxidant activity against pulmonary fibrosis^14^ and nephrotoxicity^15^. Furthermore, it exerted anti-apoptotic and nephroprotective effects in renal ischemia/reperfusion injury in rats^16^. Given these previously reported studies, we aimed to investigate the prospective protective potential of vincamine on the liver injury in rats during CLP-induced sepsis, for the first time.

## Materials and methods

### Drugs and chemicals

Vincamine was obtained from Sigma Aldrich (#1617-90-9, Sigma-Aldrich, Inc, St Louis, MO, USA). It was taken orally in 0.5% carboxymethyl cellulose (CMC) as a suspension.

### Experimental animals

Animal care and study protocols were assented (Approval No.: ES07/2021) and performed in accordance with the guidelines and regulations by Minia University's Research Ethics Committee and Experimental Animal Center, Egypt. Male Wistar rats (n = 40), weights ranged from 180 to 200 g, were acquired from the Research Center, Faculty of Medicine, Minia University, Minia, Egypt. Animals were contained in cages separately, fed with available pellets, drank fresh water, and lived in an acceptable condition (12 h of light/dark cycles) during the experiment.

### Induction of sepsis

To initiate sepsis in animals, the CLP model was utilized as described before^17^. In brief, rats’ abdominal walls were shaved and cleaned with a 10% povidone-iodine solution, followed by intraperitoneal (i.p.) injection of xylazine (10 mg/kg) and ketamine (100 mg/kg) to induce anesthesia^18^. A cut was performed in a specific part of the abdomen (lower left quadrant), the cecum was obtained and ligated with a 0.3-mm silk surgical suture thread, and the cecum was punctuated twice with an 18-guage syringe needle in the ligated portion. An approximate equal amount of cecum length (75%) was ligated in all animals to avoid variations. The sham group experienced the same conditions without obtaining and ligating of the cecum.

### Experimental design

A total of fifty Wistar rats (male) were aimlessly dispersed into five groups (n = 10) as follows:

Group I (Sham group, n = 10): rats received oral administration of 0.5% CMC by gavage for 4 consecutive days, whereas only the abdominal wall was incised on day 4^19^.

Group II (Vincamine group, n = 10): rats received oral administration of Vincamine (40 mg/kg) by gavage dissolved in 0.5% CMC daily during the entire experiment^15^.

Group III (CLP group, n = 10): rats received oral administration of 0.5% CMC by gavage for 4 consecutive days, and the CLP procedure was established on day 4.

Group IV (CLP/Vincamine group, n = 10): rats received oral administration of Vincamine (40 mg/kg) by gavage, and the CLP procedure was established on day 4.

Group V (CLP/Vitamin C, n = 10): rats were administered vitamin C intraperitoneally (single dose = 200 mg/kg) on day 4, after the CLP induction^19^.

### Sample preparation

In the fifth day, i.p. injection of urethane (25% in a dose of 1.6 g/kg) was utilized to anaesthetize rats^20^. Blood samples were collected, the serum was obtained and stored at −20 °C for liver enzymes analysis. Liver tissues were dissected and diced into three sections. For histological examinations, the first section was fixed in 10% formaldehyde. The second section was kept at −80 °C for further western blotting analysis. The third section was subjected to liquid nitrogen instantly, then stored in −20 °C for ELISA examinations.

### Evaluation of liver function tests

Liver serum enzymes such as alanine transaminase (ALT) and aspartate transaminase (AST) were evaluated to examine liver function tests according to manufacturer’s instruction (#11533, Biosystems, Barcelona, Spain) and (#11531, Biosystems, Barcelona, Spain), respectively.

### Evaluation of oxidative stress condition

Hepatic tissue samples, using the corresponding investigating kit, were used to detect the levels of reduced glutathione (GSH) (#GR2511, Biodiagnositic, Giza, Egypt), malondialdehyde (MDA) (#MD 2529, Biodiagnositic), and superoxide dismutase (SOD) (# SD 2521, Biodiagnositic), according to the manufacturer’s instructions.

### ELISA techniques

Hepatic levels of IL-6, IL-1β, and TNFα inflammatory mediators were examined according to the manufacturer’s instructions, utilizing (#E EL R0015, Elabscience, Houston, Texas, USA), (#E-EL-R0012, Elabscience, Houston, Texas, USA), and (#E-EL-R0019, Elabscience, Houston, Texas, USA), respectively. Also, hepatic cleaved caspase 3, bax, and bcl2 contents were evaluated utilizing (#MBS018987, MyBioSource, CA, USA), (#MBS935667, MyBioSource, CA, USA), and (#MBS2515143, MyBioSource, CA, USA), respectively.

### Western blotting analysis

The lysis buffer (10 mM Tris, 1 mM EDTA, 100 mM NaCl, and 0.5% Triton X-100 buffer) was used to mix and homogenate hepatic tissue samples^21,22^. 30 μg of total protein homogenates were added to a 2-mercaptoethanol loading buffer for 5 min, then combined with 12% sodium dodecyl sulfate–polyacrylamide gel electrophoresis (SDS-PAGE) and run for 2 h at 100 V***.*** After that, blotted proteins on polyvinylidene fluoride (PVDF) membranes were blocked for an hour in a Tris-buffered Saline (TBS-T) blocking solution, containing 5% (w/v) non-fat milk and 0.05% Tween-20. A Bio-Rad Trans-Blot SD Cell apparatus (Bio-Rad, Hercules, CA, USA) was used for electrophoresis and electroblotting. The blots were cut prior to hybridization with antibodies during blotting. Then, proteins were incubated with primary antibodies, including anti-Nuclear factor erythroid 2-related factor 2 (anti-Nrf-2) antibody (ab92946, Abcam, UK), anti-Kelch-like ECH-associated protein (anti-Keap-1) antibody (ab227828, Abcam, UK), and β-actin antibody (Santa Cruz Biotechnology, Santa Cruz, CA), overnight at 4 °C. As a secondary antibody, horseradish peroxidase-conjugated polyclonal immunoglobulin (1:5000) (#7074**,** Cell Signaling Technology Inc., MA, USA) was used in a blocking buffer solution. Immunoreactive proteins were detected using a Chemiluminescence kit (GE Healthcare, Little Chalfont, UK) with a luminescent image analyzer (LAS-4000, Fujifilm Co., Tokyo, Japan), according to the manufacturer’s instructions. Then, the Image Processing and Analysis Java (ImageJ, 1.8.0_172) program was used to determine the densitometric analysis. β-actin levels were used as an internal control to normalize data.

### Histological examination

Liver tissues were fixed in a neutral buffered formalin solution (10%) and processed, followed by hematoxylin and eosin (H&E) staining^23^. Using a microscope with a high-quality digital camera (Olympus, Tokyo, Japan), images were taken, and sections were evaluated. The histological examination involved an evaluation of centrilobular ballooning and degeneration, centrilobular hepatocyte necrosis, and the existence of inflammatory cell infiltration. Tissue injury was expressed as scores as follows: 0; normal, 1; slightly damaged (less than 10% of hepatocytes in the centrilobular area), 2; moderately damaged (10–50% of hepatocytes in the centrilobular area), and 3; markedly damaged (more than 50% of hepatocytes in the centrilobular area)^24^.

### Statistical analysis

For statistics, Graph Pad Prism 7 (Graph Pad Software, San Diego, California, USA) was utilized. The data were considered significant at *p* < 0.05 after using the one-way analysis of variance (ANOVA), followed by Tukey’s post hoc statistical test. The results were presented as mean ± standard deviation (SD) (n = 10).

### Ethical approval

Animal care and study protocols were preceded in accordance with ARRIVE guidelines and assented (Approval No.: ES07/2021) by Minia University's Research Ethics Committee and Experimental Animal Center, Egypt.

## Results

### Assessment of hepatic function

**Figure 1:**
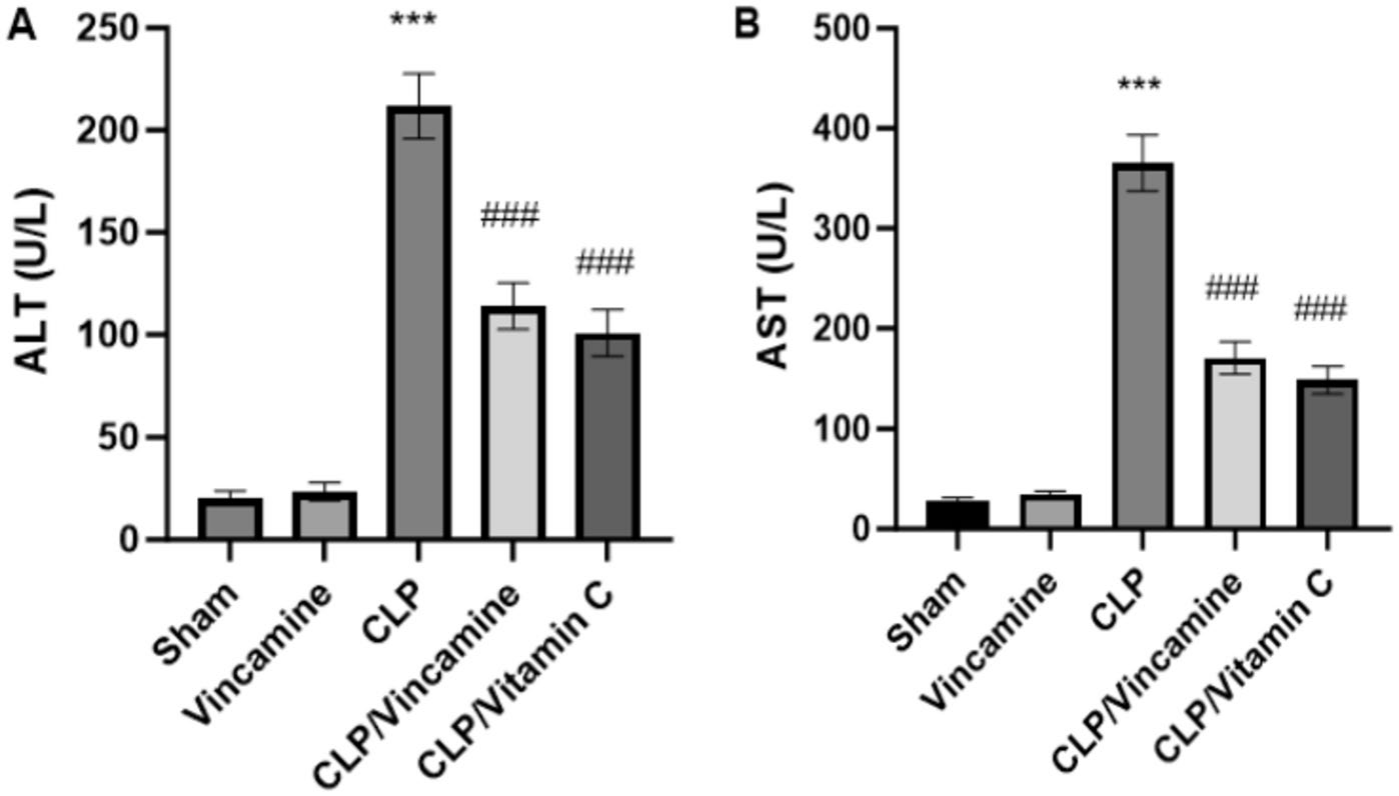
Serum levels of ALT (**A**) and AST (**B**) in different groups (n = 10). Bars represent mean ± SD. Significant difference was analyzed by one-way ANOVA test, where ***; *p* < 0.001, compared to sham group, and ###; *p* < 0.001, compared to CLP group.

Hepatic function tests were examined during CLP induction with and without vincamine pre-treatment, as demonstrated in Fig. 1. ALT and AST serum levels were significantly increased (P < 0.001) to 211.8 ± 15.79 U/L and 365.3 ± 28.1 U/L, respectively, following CLP induction, relative to sham group; however, vincamine pretreated group significantly declined (P < 0.001) serum levels of ALT and AST to 113.9 ± 11.41 U/L and 170.45 ± 15.98 U/L, respectively, compared to CLP group. Vitamin C was used as a positive control.

### Measurement of oxidative stress

**Figure 2:**
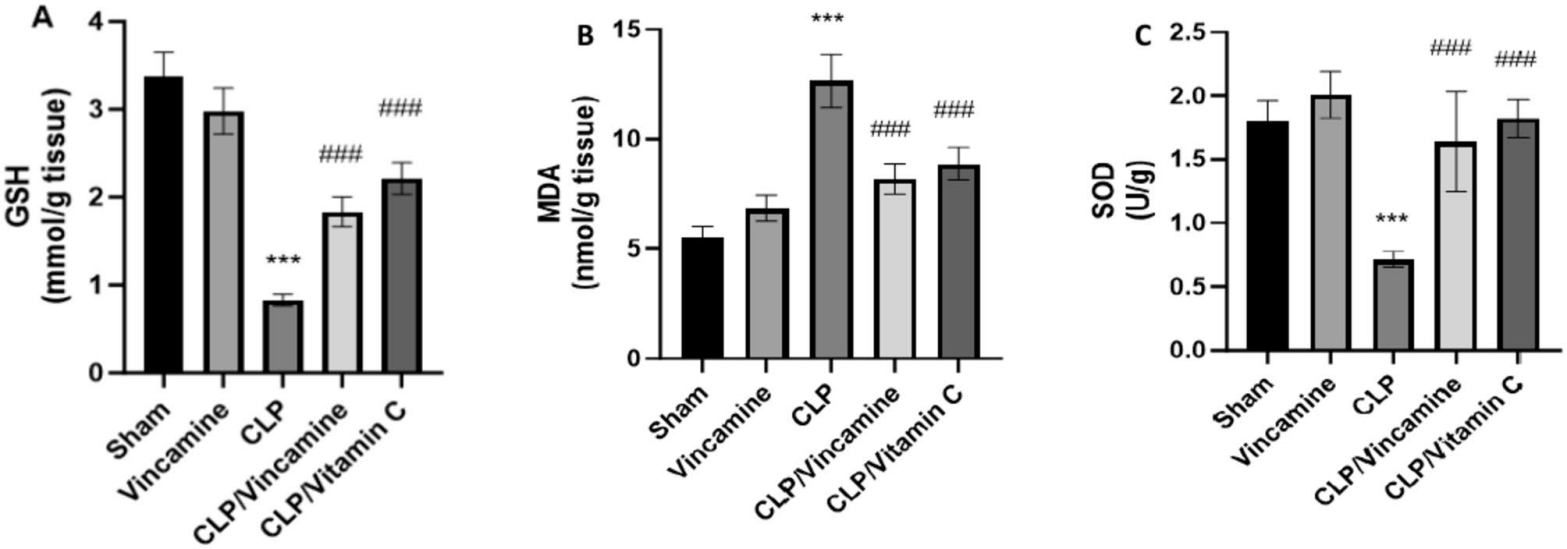
Hepatic tissue levels of GSH (**A**), MDA (**B**), and SOD (**C**) in different groups (n = 10). Bars represent mean ± SD. Significant difference was analyzed by one-way ANOVA test, where ***; *p* < 0.001, compared to sham group, and ###; *p* < 0.001, compared to CLP group.

To assess the oxidative stress condition during CLP induction with and without vincamine pre-treatment, hepatic GSH, SOD, and MDA contents were assessed. As shown in Fig. 2A–C, hepatic tissue levels of GSH and SOD were notably (P < 0.001) suppressed in CLP-induced group to 0.83 ± 0.06 mmol/g and 0.71 ± 0.06 U/g, respectively, compared to sham group. However, vincamine pre-treatment modulated these results, whereas GSH and SOD levels were notably elevated (P < 0.001) to 1.85 ± 0.17 mmol/g and 1.64 ± 0.39 U/g, respectively, compared to CLP-induced group. Contrarily, MDA levels were notably elevated (P < 0.001) during CLP induction to 12.63 ± 1.19 nmol/g, relative to sham group, while vincamine notably decreased.

MDA levels to 8.17 ± 0.71 nmol/g, compared to CLP group, as shown in Fig. 2B. Vitamin C was used as a positive control.

### Evaluation of inflammatory and apoptotic markers

**Figure 3:**
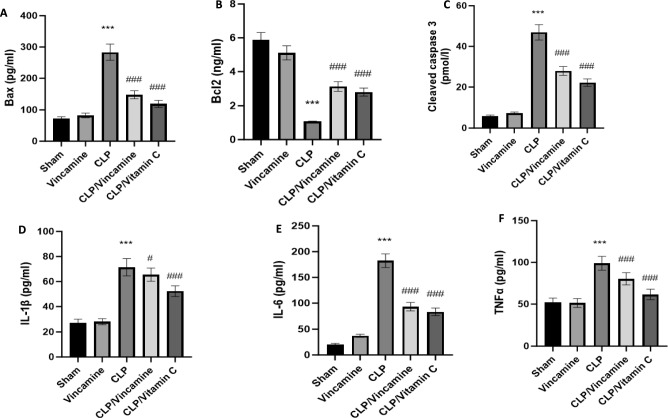
Hepatic tissue levels of bax (**A**), bcl2 (**B**), cleaved caspase 3 (**C**), IL-1β (**D**), IL-6 (**E**), and TNFα (**F**) in different groups (n = 10). Bars represent mean ± SD. Significant difference was analyzed by one-way ANOVA test, where ***; *p* < 0.001, compared to sham group, and ###; *p* < 0.001, compared to CLP group.

To evaluate inflammatory conditions in the present study, TNFα, IL-6, and IL-1β levels were assessed during CLP-induction with and without vincamine pretreatment. As shown in Fig. 3A–C, hepatic tissue levels of TNFα, IL-6, and IL-1β were notably (P < 0.001) elevated to 98.93 ± 8.27 pg/ml, 182.41 ± 13.29 pg/ml, and 71.42 ± 6.95 pg/ml, respectively, compared to sham group. While vincamine treated group exhibited significant attenuation (P < 0001) in these levels to 80.29 ± 7.36 pg/ml, 93.17 ± 8.24 pg/ml, and 65.47 ± 5.28 pg/ml for TNFα, IL-6, and IL-1β, respectively, compared to CLP-induced group.

Regarding the apoptotic status during CLP induction with and without vincamine treatment, the hepatic tissue levels of cleaved caspase 3, bax, and bcl2 were examined.

As shown in Fig. 3D–F, the tissue levels of cleaved caspase 3, bax, and bcl2 were significantly modulated (P < 0.001) to 46.92 ± 3.82 pmol/l, 283.29 ± 25.91 pg/ml, and 1.07 ± 0.018 ng/ml, respectively, during CLP induction relative to sham group. However, vincamine pretreatment significantly attenuated (P < 0.001) cleaved caspase 3 and bax levels to 27.92 ± 2.17 pmol/l and 147.27 ± 12.81 pg/ml, respectively, compared to CLP-induced group. In addition, a significant increase (P < 0.001) in bcl2 levels was observed in vincamine-treated group to 3.12 ± 0.23 ng/ml, compared to the CLP-induced group.

### Western blotting

**Figure 4:**
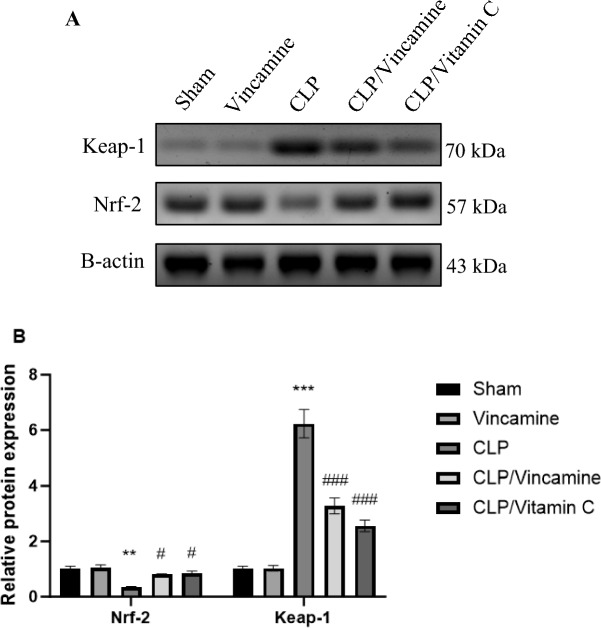
Effect of Vincamine on the hepatic expression of Nrf-2 and Keap-1 proteins. (**A**) Representative western blots of Nrf-2 and Keap-1 proteins in different groups. (**B**) Expression of Nrf-2 and Keap-1 proteins densitometrically, using bands in (**A**) after normalization to the corresponding internal control β-actin as fold change relative to that of sham control rats. Bars represent mean ± SD. Significant difference was analyzed by two-way ANOVA test, where **; *p* < 0.01, ***; *p* < 0.001, compared to sham group, and #; p < 0.05, ###; *p* < 0.001 compared to CLP group.

Hepatic protein expression levels of keap-1 and Nrf-2 were evaluated. As shown in Fig. 4 and the supplementary file, Keap-1 protein expression was notably (P < 0.001) elevated in CLP-induced group relative to sham group, while vincamine treatment inhibited (P < 0.001) its expression compared to CLP group. On the other hand, Nrf2 protein expression was suppressed (P < 0.01) in CLP-induced group relative to sham group, while vincamine treatment significantly increased (P < 0.05) its protein expression compared to CLP-induced group.

### Histopathological examination

**Figure 5:**
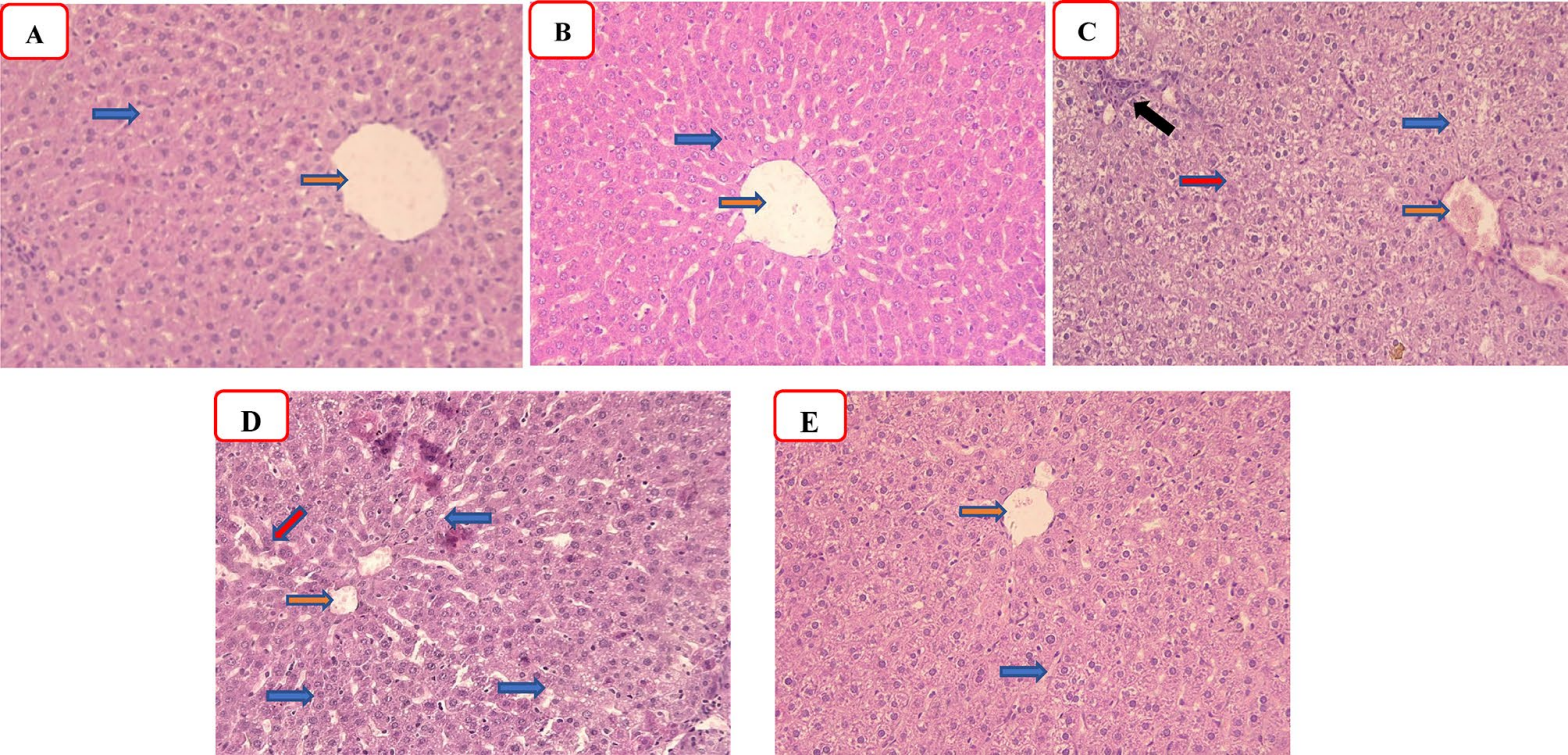
Representative photomicrographs of hepatic tissues (H&E, ×200) of the sham group (**A**), R group (**B**), CLP group (**C**), CLP/R group (**D**), and CLP + vitamin C (**E**). Orange arrow: hepatic central vein, blue arrow: hepatocytes, black arrow: inflammatory cell infiltrate, and red arrow: unicellular necrosis.

As demonstrated in Fig. 5 and Table 1, the effect of vincamine on the CLP-induced hepatic histopathological alterations was investigated. Both sham and vincamine groups showed normal hepatic lobules composed of normal hepatocytes (blue arrow) arranged around normal central vein (red arrow) with the absence of periportal inflammation (Fig. 5A, B). The CLP group demonstrated marked areas of ballooning and cytoplasmic vacuolations (blue arrow), centrilobular necrosis with a dilated congested central vein (red arrow), and a marked periportal inflammatory cell infiltrate (black arrow) (Fig. 5C). CLP/vincamine-treated group showed mild hepatocyte ballooning and cytoplasmic vacuolations with absent necrosis (blue arrow), mild inflammatory cell infiltrate, and mild central vein and sinusoidal congestion (red arrow) (Fig. 5D). CLP/vitamin C group demonstrated minimal centrilobular hepatocyte degeneration and inflammation with the absence of necrosis and mild central vein and sinusoidal congestion (Fig. 5E).

**Table 1:** Histological scoring of different groups.

Groups	Sham	Vincamine	CLP	CLP/vincamine	CLP/vitamin C
Inflammation	0	0	3	1	1
Necrosis	0	0	3	1	1
Ballooning degeneration	0	0	2	2	1
Total scoring	0	0	8*	4^**#**^	3^#^

Significant difference was analyzed by one-way ANOVA test followed by Kruskal–Wallis statistical test, where P < 0.001.

*Relative to sham group.

^#^Relative to CLP group.

## Discussion

Sepsis is a pathological and biochemical disorder induced by numerous infections, leading to critical illness and a high mortality rate worldwide^25^. It is a life-threatening condition with multiple organ dysfunction caused by an impairment of the host immune response to infection^26^. Liver dysfunction during sepsis contributes primarily to poor prognosis, multiple organ failure, and an elevated incidence of death^27^. The underlying mechanism of sepsis-induced hepatic failure is notably complex; interestingly, the elevated oxidative stress condition associated with the systemic hyperinflammatory reaction contributes to hepatic injury^28^. Therefore, screening for new therapeutic agents that show antioxidant and anti-inflammatory activity is needed to manage sepsis^29,30^.

In the present study, we evaluated the effect on liver enzymes during CLP-induced sepsis with and without vincamine treatment. Our findings revealed that ALT and AST serum levels were elevated during CLP induction; however, vincamine pre-treatment notably decreased their levels, indicating its hepato-protective activity against liver injury induced by CLP. In addition, the histopathological examinations came to confirm our analysis, whereas CLP increased ballooning, necrosis, and inflammation within the hepatocyte, while vincamine pre-treatment ameliorated these CLP-induced alterations. This paved the way for further analysis of the hepato-protective activity of vincamine during CLP induction.

We further analyzed the oxidative stress condition and the inflammatory status during CLP induction with and without vincamine pre-treatment since there is a cross-communication between oxidative stress and inflammation^31–33^. The cell's antioxidant defense system, including GSH and SOD, was suppressed during CLP induction, while MDA levels, a commonly used marker for evaluating oxidative stress^34–36^, were elevated during CLP induction. However, vincamine pre-treatment exhibited marked antioxidant activity, whereas GSH and SOD hepatic tissues were elevated and MDA levels were suppressed, indicating the antioxidant potentiality of vincamine during CLP-induced sepsis.

Keap-1/Nrf-2 signaling system plays a significant role in the maintenance of cellular hemostasis under stress condition. Normally, the transcription factor Nrf-2 is located in the cytoplasm and is sequestered and degraded by Keap-1 protein during physiological condition^37^. However, under oxidative stress, modifications of Keap-1 disrupt this interaction, allowing Nrf-2 to accumulate and translocate to the nucleus. In the nucleus, Nrf-2 binds to antioxidant response elements in the DNA, promoting the expression of various antioxidant and cytoprotective genes. These genes encode for enzymes and proteins that neutralize reactive oxygen species, repair damaged DNA, and restore redox homeostasis^38,39^. Additionally, TNFα, a central pro-inflammatory mediator involved in the pathogenesis of some inflammatory and autoimmune diseases, was notably reported to suppress Nrf-2 expression^40,41^. Also, a previous study reported that the upregulation of Keap-1 protein was following TNFα elevation, indicating that TNFα increases Keap-1 mRNA expression^31^. Contrarily, it is known that oxidative stress can trigger the activation of Nrf-2^42^. However, it was suggested that oxidative stress in normal physiological conditions could activate Nrf-2 signaling, while persistent oxidative stress status results in the activation of a chronic inflammatory response and leads to the activation of TNFα signaling and inhibition of Nrf-2 expression^42^.

In the present study, we evaluated the inflammatory condition-mediated Nrf-2 dysregulation. Our findings revealed that TNFα, IL-6, and IL-1β, pro-inflammatory cytokines, were elevated during CLP induction in addition to the downregulation of Nrf-2 protein and upregulation of Keap-1 protein. While vincamine pre-treatment exhibited notable attenuation of the inflammatory reaction through the inhibition of TNFα, IL-6, IL-1β, and Keap-1 expression, and activating the protein expression of Nrf-2, strongly suggesting the anti-inflammatory potential of vincamine during the hepatic inflammatory condition-induced by CLP.

Furthermore, the present study evaluated the apoptotic status of liver tissues during CLP induction with and without vincamine pre-treatment. Hepatic tissue levels of pro-apoptotic factors such as bax and cleaved caspase 3 proteins were elevated during CLP induction, while anti-apoptotic bcl2 protein tissue levels were inhibited, which suggests the initiation of apoptosis. However, vincamine pre-treatment modulated these levels, resulting in attenuation of hepatic apoptosis, suggesting the anti-apoptotic activity of vincamine during CLP-induced sepsis.

Therefore, vincamine showed hepato-protective activity during CLP-induced sepsis via the cross-connection of antioxidant and anti-inflammatory activities.

## Conclusion

Vincamine exhibited hepato-protective potential during CLP-induced sepsis via the cross-connection of antioxidant, anti-inflammatory and anti-apoptotic activities. It ameliorated hepatic oxidative stress by reducing MDA content and enhanced antioxidant status by increasing SOD activity and GSH content. Moreover, vincamine attenuated the inflammation by modulating TNFα/IL-6/IL-1β/Nrf-2/Keap-1 signaling pathway and suppressed the apoptosis by regulating bax/bcl2/cleaved caspase 3 signaling pathway.

### Supplementary Information

Supplementary Information.

## Data Availability

All data generated or analyzed during this study are included in this published article and its supplementary information files.

## References

[1] Siempos, I. I. *et al.* Cecal ligation and puncture-induced sepsis as a model to study autophagy in mice. *J. Vis. Exp.* **84**, e51066. 10.3791/51066 (2014). 10.3791/51066 PMC4122027 24561344

[2] Bone, R. C., Grodzin, C. J. & Balk, R. A. Sepsis: A new hypothesis for pathogenesis of the disease process. *Chest* **112**(1), 235–243 (1997). 9228382 10.1378/chest.112.1.235

[3] Yan, J., Li, S. & Li, S. The role of the liver in sepsis. *Int. Rev. Immunol.* **33**(6), 498–510. 10.3109/08830185.2014.889129 (2014). 24611785 10.3109/08830185.2014.889129 PMC4160418

[4] Iwashyna, T. J., Cooke, C. R., Wunsch, H. & Kahn, J. M. Population burden of long-term survivorship after severe sepsis in older Americans. *J. Am. Geriatr. Soc.* **60**(6), 1070–1077 (2012). 22642542 10.1111/j.1532-5415.2012.03989.x PMC3374893

[5] Gaieski, D. F., Edwards, J. M., Kallan, M. J. & Carr, B. G. Benchmarking the incidence and mortality of severe sepsis in the United States. *Crit. Care Med.* **41**(5), 1167–1174 (2013). 23442987 10.1097/CCM.0b013e31827c09f8

[6] Deitch, E. A. Rodent models of intra-abdominal infection. *Shock* **24**, 19–23 (2005). 16374368 10.1097/01.shk.0000191386.18818.0a

[7] Raven, K. Rodent models of sepsis found shockingly lacking. *Nat. Med.* **18**(7), 998 (2012). 22772539 10.1038/nm0712-998a

[8] Vincent, J.-L. *et al.* Effects of drotrecogin alfa (activated) on organ dysfunction in the PROWESS trial. *Crit. Care Med.* **31**(3), 834–840 (2003). 12626993 10.1097/01.CCM.0000051515.56179.E1

[9] Arfaras-Melainis, A. *et al.* Heart failure and sepsis: Practical recommendations for the optimal management. *Heart Fail. Rev.* **25**, 183–194 (2020). 31227942 10.1007/s10741-019-09816-y

[10] Ren, Y. *et al.* Vincamine, from an antioxidant and a cerebral vasodilator to its anticancer potential. *Bioorgan. Med. Chem.*10.1016/j.bmc.2023.117439 (2023). 10.1016/j.bmc.2023.117439 PMC10530545 37579526

[11] Alaaeldin, R., Eisa, Y. A., El-Rehany, M. A. & Fathy, M. Vincamine alleviates intrahepatic cholestasis in rats through modulation of NF-kB/PDGF/klf6/PPARgamma and PI3K/Akt pathways. *Naunyn Schmiedebergs Arch. Pharmacol.*10.1007/s00210-024-03119-2 (2024). 38761209 10.1007/s00210-024-03119-2 PMC11449999

[12] Lakshmi, V. *et al.* Vincamine and Vindoline from *Catharanthus roseus* linn. protects the gastric mucosa of gastric ulcer in rats. *Pharmacologia* **4**, 243–248 (2013). 10.5567/pharmacologia.2013.243.248

[13] Wu, L., Ye, M. & Zhang, J. Vincamine prevents lipopolysaccharide induced inflammation and oxidative stress via thioredoxin reductase activation in human corneal epithelial cells. *Am. J. Transl. Res.* **10**(7), 2195 (2018). 30093956 PMC6079141

[14] Alaaeldin, R. *et al.* Vincamine ameliorates epithelial-mesenchymal transition in bleomycin-induced pulmonary fibrosis in rats; targeting TGF-β/MAPK/Snai1 pathway. *Molecules* **28**(12), 4665 (2023). 37375218 10.3390/molecules28124665 PMC10303541

[15] El-Sayed, R. M., AboElGheit, R. E. & Badawi, G. A. Vincamine protects against cisplatin induced nephrotoxicity via activation of Nrf2/HO-1 and hindering TLR4/ IFN-γ/CD44 cells inflammatory cascade. *Life Sci.* **272**, 119224. 10.1016/j.lfs.2021.119224 (2021). 33610575 10.1016/j.lfs.2021.119224

[16] Fawzy, M. A. *et al.* Vincamine modulates the effect of pantoprazole in renal ischemia/reperfusion injury by attenuating MAPK and apoptosis signaling pathways. *Molecules* **27**(4), 1383. 10.3390/molecules27041383 (2022). 35209172 10.3390/molecules27041383 PMC8879001

[17] Deitch, E. A. Animal models of sepsis and shock: A review and lessons learned. *Shock* **10**(6), 442–443 (1998). 9466467 10.1097/00024382-199812000-00011

[18] Wu, G.-J., Lin, Y.-W., Chuang, C.-Y., Tsai, H.-C. & Chen, R.-M. Liver nitrosation and inflammation in septic rats were suppressed by propofol via downregulating TLR4/NF-κB-mediated iNOS and IL-6 gene expressions. *Life Sci.* **195**, 25–32 (2018). 29307523 10.1016/j.lfs.2018.01.005

[19] Abdelnaser, M., Alaaeldin, R., Attya, M. E. & Fathy, M. Hepatoprotective potential of gabapentin in cecal ligation and puncture-induced sepsis; targeting oxidative stress, apoptosis, and NF-kB/MAPK signaling pathways. *Life Sci.* **320**, 121562. 10.1016/j.lfs.2023.121562 (2023). 36907325 10.1016/j.lfs.2023.121562

[20] Abdelzaher, W. Y. *et al.* The protective effect of fenofibrate, triptorelin, and their combination against premature ovarian failure in rats. *Naunyn Schmiedebergs Arch. Pharmacol.* **394**(1), 137–149. 10.1007/s00210-020-01975-2 (2021). 32924068 10.1007/s00210-020-01975-2

[21] Abdel-Hamid, N. M. *et al.* Identification of chemo and radio-resistant sub-population of stem cells in human cervical cancer HeLa cells. *Cancer Invest.* **39**(8), 661–674 (2021). 34076552 10.1080/07357907.2021.1931875

[22] Wang, F. *et al.* CD24+ SSEA4+ cells in ovarian carcinoma cells demonstrated the characteristics as cancer stem cells. *J. Cancer Sci. Ther.* **9**, 343–352 (2017). 10.4172/1948-5956.1000440

[23] Bancroft, J.D. & Gamble, M. *Theory and Practice of Histological Techniques*. (Elsevier Health Sciences, 2008).

[24] Naiki-Ito, A. *et al.* Gap junction dysfunction reduces acetaminophen hepatotoxicity with impact on apoptotic signaling and connexin 43 protein induction in rat. *Toxicol. Pathol.* **38**(2), 280–286 (2010). 20097795 10.1177/0192623309357951

[25] Vincent, J.-L. *et al.* Assessment of the worldwide burden of critical illness: The intensive care over nations (ICON) audit. *Lancet Respir. Med.* **2**(5), 380–386 (2014). 24740011 10.1016/S2213-2600(14)70061-X

[26] Singer, M. *et al.* The third international consensus definitions for sepsis and septic shock (Sepsis-3). *Jama* **315**(8), 801–810 (2016). 26903338 10.1001/jama.2016.0287 PMC4968574

[27] Fuhrmann, V. *et al.* Hepatopulmonary syndrome in patients with hypoxic hepatitis. *Gastroenterology* **131**(1), 69–75 (2006). 16831591 10.1053/j.gastro.2006.04.014

[28] Liu, X. *et al.* Pterostilbene alleviates polymicrobial sepsis-induced liver injury: Possible role of SIRT1 signaling. *Int. Immunopharmacol.* **49**, 50–59 (2017). 28550734 10.1016/j.intimp.2017.05.022

[29] Abdelnaser, M., Alaaeldin, R., Attya, M. E. & Fathy, M. Modulating Nrf-2/HO-1, apoptosis and oxidative stress signaling pathways by gabapentin ameliorates sepsis-induced acute kidney injury. *Naunyn-Schmiedeberg’s Arch. Pharmacol.* **4**, 1–12 (2023). 10.1007/s00210-023-02650-y PMC10791735 37548662

[30] Alaaeldin, R., Mustafa, M., Abuo-Rahma, G. E. D. A. & Fathy, M. In vitro inhibition and molecular docking of a new ciprofloxacin-chalcone against SARS-CoV-2 main protease. *Fund. Clin. Pharmacol.* **36**(1), 160–170 (2022). 10.1111/fcp.12708 PMC8444764 34268806

[31] Ren, L. *et al.* Curcumin upregulates the Nrf2 system by repressing inflammatory signaling-mediated Keap1 expression in insulin-resistant conditions. *Biochem. Biophys. Res. Commun.* **514**(3), 691–698. 10.1016/j.bbrc.2019.05.010 (2019). 31078267 10.1016/j.bbrc.2019.05.010

[32] Alaaeldin, R., Abuo-Rahma, G.E.-D.A., Zhao, Q.-L. & Fathy, M. Modulation of apoptosis and epithelial-mesenchymal transition E-cadherin/TGF-β/Snail/TWIST pathways by a new ciprofloxacin chalcone in breast cancer cells. *Anticancer Res.* **41**(5), 2383–2395 (2021). 33952463 10.21873/anticanres.15013

[33] Fathy, M., Okabe, M., Saad Eldien, H. M. & Yoshida, T. AT-MSCs antifibrotic activity is improved by eugenol through modulation of TGF-beta/Smad signaling pathway in rats. *Molecules*10.3390/molecules25020348 (2020). 31952158 10.3390/molecules25020348 PMC7024200

[34] Gaweł, S., Wardas, M., Niedworok, E. & Wardas, P. Malondialdehyde (MDA) as a lipid peroxidation marker. *Wiad Lek* **57**(9–10), 453–455 (2004). 15765761

[35] Alaaeldin, R., Ali, F. E., Bekhit, A. A., Zhao, Q.-L. & Fathy, M. Inhibition of NF-kB/IL-6/JAK2/STAT3 pathway and epithelial-mesenchymal transition in breast cancer cells by azilsartan. *Molecules* **27**(22), 7825 (2022). 36431925 10.3390/molecules27227825 PMC9693603

[36] Alaaeldin, R. *et al.* A new EGFR inhibitor from *Ficus benghalensis* exerted potential anti-inflammatory activity via Akt/PI3K pathway inhibition. *Curr. Issues Mol. Biol.* **44**(7), 2967–2981 (2022). 35877429 10.3390/cimb44070205 PMC9324879

[37] Silva-Palacios, A., Königsberg, M. & Zazueta, C. Nrf2 signaling and redox homeostasis in the aging heart: A potential target to prevent cardiovascular diseases?. *Ageing Res. Rev.* **26**, 81–95. 10.1016/j.arr.2015.12.005 (2016). 26732035 10.1016/j.arr.2015.12.005

[38] Bellezza, I., Giambanco, I. & Minelli, A. Nrf2-Keap1 signaling in oxidative and reductive stress. *Biochim. Biophys. Acta BBA Mol. Cell Res.* **1865**, 721–733 (2018). 10.1016/j.bbamcr.2018.02.010 29499228

[39] Fawzy, M. A., Nasr, G., Ali, F. E. M. & Fathy, M. Quercetin potentiates the hepatoprotective effect of sildenafil and/or pentoxifylline against intrahepatic cholestasis: Role of Nrf2/ARE, TLR4/NF-κB, and NLRP3/IL-1β signaling pathways. *Life Sci.* **314**, 121343. 10.1016/j.lfs.2022.121343 (2023). 36592787 10.1016/j.lfs.2022.121343

[40] Liu, G.-H., Qu, J. & Shen, X. NF-κB/p65 antagonizes Nrf2-ARE pathway by depriving CBP from Nrf2 and facilitating recruitment of HDAC3 to MafK. *Biochim. Biophys. Acta (BBA) Mol. Cell Res.* **1783**(5), 713–727 (2008). 10.1016/j.bbamcr.2008.01.002 18241676

[41] Alaaeldin, R. *et al.* Azilsartan modulates HMGB1/NF-κB/p38/ERK1/2/JNK and apoptosis pathways during renal ischemia reperfusion injury. *Cells* **12**(1), 185 (2023). 36611978 10.3390/cells12010185 PMC9818604

[42] Li, W. & Kong, A. N. Molecular mechanisms of Nrf2-mediated antioxidant response. *Mol. Carcinogen. (published in cooperation with the University of Texas MD Anderson Cancer Center)* **48**(2), 91–104 (2009). 10.1002/mc.20465 PMC2631094 18618599

