# Supplementary material for: Vincamine exerts hepato-protective activity during colon ligation puncture-induced sepsis by modulating oxidative stress, apoptosis, and TNFα/Nrf-2/Keap-1 signaling pathways
Source: Sci Rep. 2024 Aug 23;14:19572. doi: 10.1038/s41598-024-69729-1 (PMC11341710; doi:10.1038/s41598-024-69729-1)

Keap-1
(70 kDa)

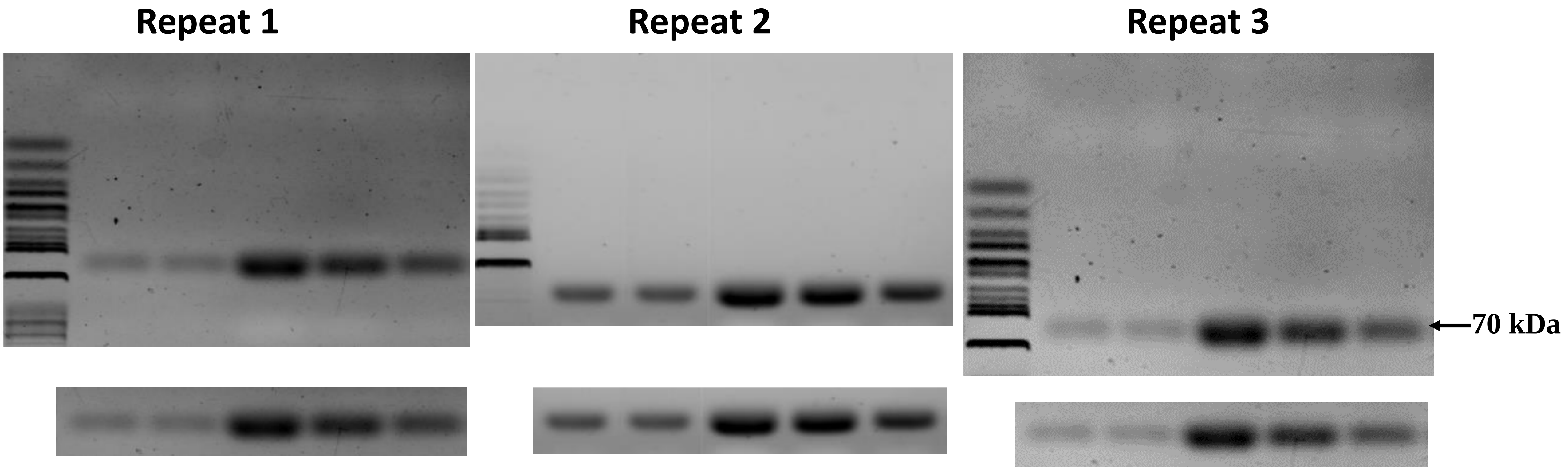

Nrf2
(57 kDa)

Repeat 1 Repeat 2 Repeat 3

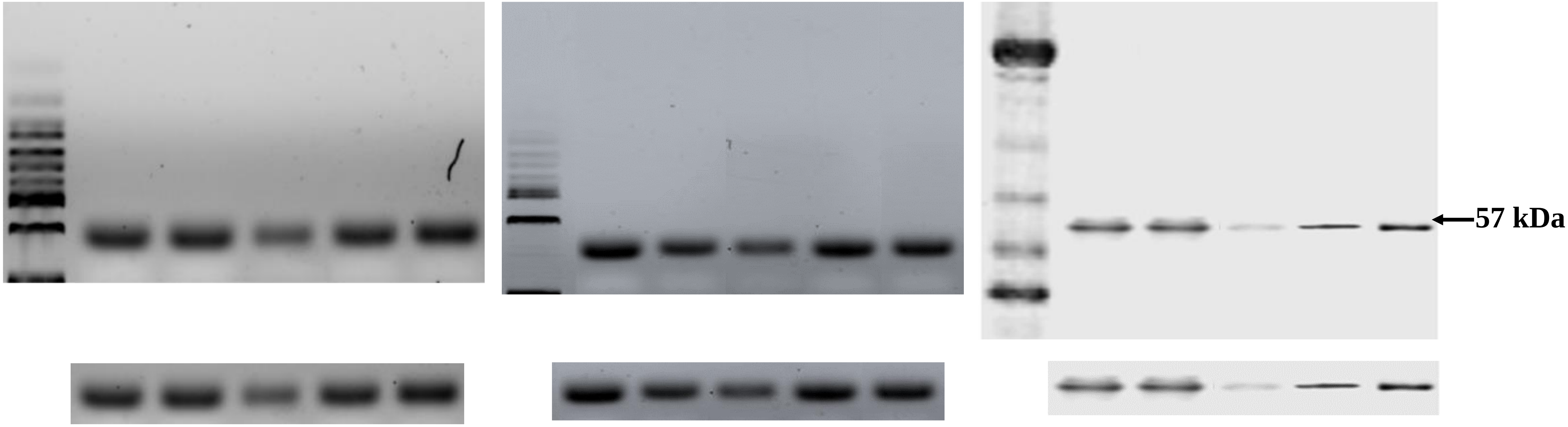

B-actin
(43 kDa)

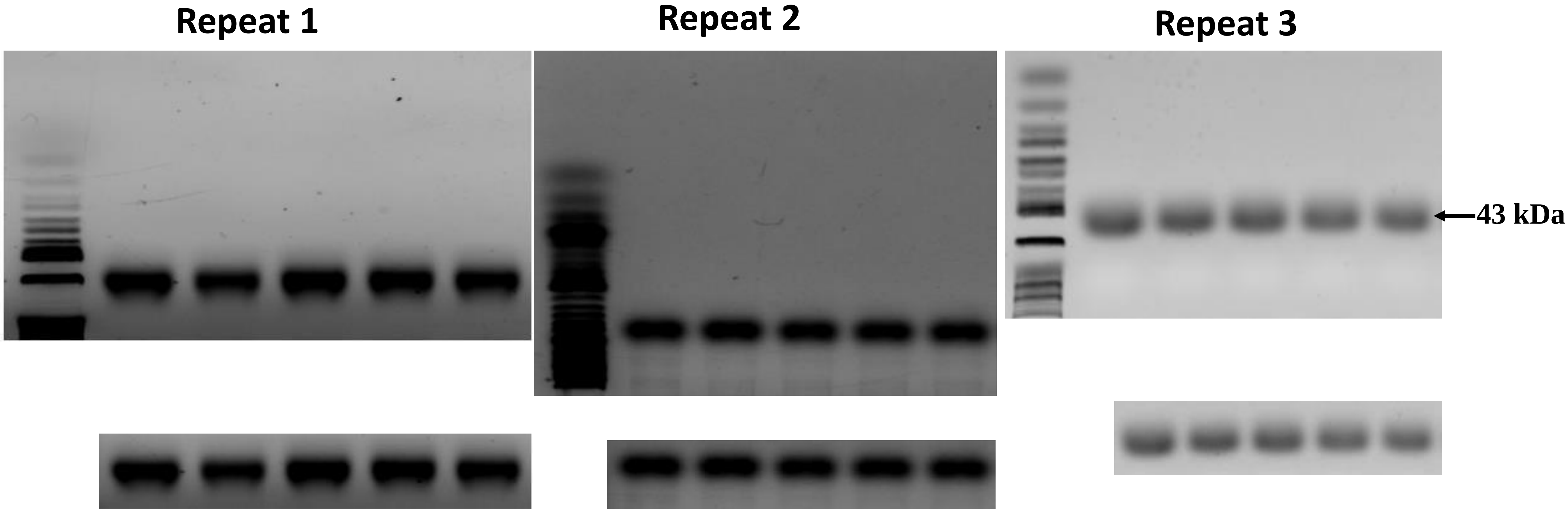

Supplement: Supplementary file 1 — Supplementary Information. [file 41598_2024_69729_MOESM1_ESM.pdf]
